# Open science falling behind in the era of artificial intelligence

**DOI:** 10.3389/frma.2025.1595824

**Published:** 2025-07-24

**Authors:** Guanxiong Pei, Huajian Huang

**Affiliations:** ^1^Zhejiang Laboratory of Philosophy and Social Sciences - Laboratory of Intelligent Society and Governance, Zhejiang Lab, Hangzhou, China; ^2^Development Strategy and Cooperation Center, Zhejiang Lab, Hangzhou, China; ^3^School of Innovation and Entrepreneurship, Zhejiang University, Hangzhou, China; ^4^Entrepreneurship Development Association of Hangzhou, Hangzhou, China

**Keywords:** open science, AI for science, systematic policy innovations, public sector initiatives, governance policies

## Introduction

Generative Artificial Intelligence (AI) refers to a new generation of content generation technologies that emerged after the rise of Transformer architecture in 2017, characterized by its core technical features of “compute-intensive architecture, model-driven paradigm, and data closed-loop system” (Table 1). AI is accelerating scientific discoveries and reshaping the research process, propelling AI for science toward becoming a novel research paradigm. There is a pressing demand for open science due to these advancements, yet the development of open science lags considerably behind the AI era. This disparity arises from the loss of academic leadership and insufficient motivation to pursue openness within the industrial sector, which could hinder AI empowerment and scientific innovation. Effective intervention by the public sector and policymakers becomes crucial when the “invisible hand” fails.

**Table 1:** Stages of open science.

**Main aspects**	**Consensus-based 1.0 stage**	**Platform-based 2.0 stage**
Historical context	Pre-artificial intelligence era (prior to the publication of “Attention Is All You Need” in 2017)	The era of artificial intelligence and large generative AI models (2017 onwards)
Key features	•Conceptual consensus •Unified action •Open standardization	•Resource scaling •Model transparency •Interdisciplinary cooperation
Primary drivers	•Transparency in the research process •Reproducibility of findings •Knowledge sharing and research collaboration •Research integrity	•Huge demand for research resources such as data and computing power •Requirements for transparency and rigor in scientific foundation models and domain-specific models •Innovation through interdisciplinary integration
Key areas of openness	•Scientific publications •Research data •Educational resources •Source software and source code •Hardware •Engagement of societal actors •Dialogue with other knowledge systems •Infrastructure supported by the internet or local networks	•Computing power •Large-scale datasets and training corpora •Foundation models and domain-specific models •AI tools and training •Interdisciplinary fusion •Synergy between industry and academia •Infrastructure supported by cloud platforms
Dominant forces	•Public sector •Academia •Publishing	•Industry •Public sector •Academia

## A new stage of open science

AI technology has given rise to AI for science as a new research paradigm and pushed open science from the consensus-based 1.0 stage into the platform-based 2.0 stage (Table 1). How did this transformation occur?

First, the data and computational resources needed to train competitive scientific models are increasing rapidly, as seen in fields such as materials science (Lei et al., 2024), quantum computing (Huang et al., 2023), and weather forecasting (Conklin and Kumar, 2023), among others. It is impractical for individual research teams to independently deploy large-scale computing power and construct high-quality scientific databases and corpora. This necessitates the development of open scientific infrastructure that integrates computing power, data, and models into a unified supply system. Such infrastructure would enable the efficient organization of scientific innovation resources, enhance the overall quality and efficiency of AI resources, reduce operational costs, and minimize redundant efforts. Much like electricity or the internet, this new infrastructure will become an essential public good.

Second, responsible and trustworthy AI urgently requires a more open and transparent environment for model development. The proprietary nature of many AI models and the uncertain origins of their training data have raised concerns about the reproducibility, fairness, and reliability of AI-enabled scientific research. A majority of scientists (55%) have expressed concerns that AI model deployment may facilitate fraud (Van Noorden and Perkel, 2023). There is an urgent need to establish a more open and transparent environment for model development, providing avenues for public scrutiny, opportunities for result reproducibility and validation, and avenues for model refinement (Aspesi and Brand, 2020; Wang et al., 2023). These efforts aim to foster high-quality, replicable, and responsible research. According to research data from the Hugging Face platform, open model development is showing explosive growth trends. As of the end of August 2023, the number of pre-trained models on the platform has achieved leapfrog growth, skyrocketing from a cumulative total of 100,000 to over 300,000; the dataset scale has also expanded simultaneously, increasing from 10,000 to 58,000. This trend is equally evident in the scientific community: the Materials Project platform in materials science has made quantum mechanics calculation datasets accessible for over 154,000 inorganic compounds, thereby supporting global researchers in the development of new materials (Riebesell et al., 2023). Meanwhile, the Protein Data Bank (PDB) in structural biology has shared more than 220,000 three-dimensional protein structure datasets through standardized formats, providing essential foundational data for AI-driven drug discovery research (Liu et al., 2024).

Third, AI for science is inherently interdisciplinary. The primary motivation for the classification of disciplines lies in human cognitive limitations, and with the advancement of AI research tools, human understanding of the world will tend to be integrated. However, differences in knowledge systems and thinking habits create barriers between AI experts and domain scientists and require innovative collaboration models and the removal of walls between disciplines. This transformation fundamentally shifts research organizational paradigms from closed, fragmented small workshops to open, collaborative large platforms. Open science platforms enable AI experts to provide AI research tools and training and enable other experts to share domain-specific knowledge. This bidirectional collaborative approach aids in breaking down disciplinary barriers.

## The problem of industry dominance

Industry is becoming increasingly influential compared to academia. It dominates three key elements of AI research: computing power (Ali et al., 2025), large datasets (Hartmann and Henkel, 2020), and highly skilled researchers (Ahmed et al., 2023). According to the Artificial Intelligence Index Report 2025, industry developed 55 notable AI models. In contrast, academia released none (HAI, 2025). Furthermore, the rate at which AI doctoral graduates in the United States are migrating to industry continues to accelerate. In 2011, new AI PhDs took jobs in industry (40.9%) and academia (41.6%) in roughly equal proportions. However, by 2022, a considerably larger proportion (70.7%) chose industry compared to academia (20.0%) (Ahmed et al., 2023). With extremely high salaries and expensive computing power to back them up, technology giants around the world are attracting top talent and producing world-shaking achievements such as AlphaFold and ChatGPT. Such a profound and systemic change is taking place that the “godmother of AI” Fei-Fei Li has made an urgent appeal to US President Joe Biden for funding to prevent Silicon Valley from pricing academics out of AI research (Nix et al., 2024). Why is industry dominance problematic?

Industrial innovators may seek to erect barriers by controlling computing resources and datasets, closing off source code, and making models proprietary to maintain their competitive advantage. This closed strategy stems from the fundamental conflict between industry's pursuit of maximizing shareholder value and academia's commitment to public knowledge sharing, though concerns about potential risks introduced by model open-sourcing also play a role. For instance, releasing model weights may create risks of AI misuse (Kim et al., 2025). While such risks could be mitigated through technical approaches like federated learning (Xu et al., 2020), these safety measures might increase model development costs. After weighing the scientific value of openness against commercial risks, industry continues to prioritize proprietary control over open science principles. These entities generally lack interest in creating public scientific goods and often exclude scientists not driven by shareholder value from AI research endeavors.

Notably, open science practices are not without benefits. Taking talent acquisition as an example, after Meta open-sourced LLaMA and LLaMA 2, it attracted top AI researchers from Google DeepMind, OpenAI, and academia, with many open-source contributors eventually joining Meta's AI teams. Concurrently, over 100,000 derivative models based on LLaMA emerged on Hugging Face, fostering a robust developer ecosystem. While many companies recognize that openness can yield long-term competitive advantages—such as attracting talent, fostering synergy between industry and academia, and building robust ecosystems—they often choose closed approaches motivated by short-term commercial interests, including the protection of proprietary technologies and the pursuit of first-mover advantages. This “knowing-but-not-doing” paradox highlights the fundamental conflict between research ethics and business logic.

Lack of resources in academia and lack of motivation in industry may slow the open science process and hinder the value output of AI for science. According to the Artificial Intelligence Index Report 2024, while 65.7% of foundational models were open-source in 2023—an increase from 44.4% in 2022—the highest-performing models, such as GPT-4 and Gemini Ultra, continue to be predominantly closed-source and controlled by industry players (HAI, 2024). A survey of 1,600 scientists highlighted lack of computational resources, funding for research activities, and access to scientific platforms and high-quality data essential for AI applications (Van Noorden and Perkel, 2023). A number of important studies are hampered by the lack of access to advanced models. Large-scale and powerful AI models and tools are often proprietary, large amounts of valuable data are stored in repositories with restricted access, incompatible software formats hinder scientific collaboration, complex communication channels delay knowledge sharing, and AI tools are relatively expensive to use, with only a fraction of researchers being able to access the resources and afford the associated costs.

## Recommendations for policymakers

A systematic policy framework in two dimensions is proposed. Vertically, synergies are formed between top-down AI for science cloud infrastructure initiatives driven by the public sector and bottom-up efforts to cultivate a widespread awareness of open science. Horizontally, incentive policies act as accelerators while governance policies serve as safeguards to ensure efficient and reliable operation.

### Top-down initiatives: AI for science cloud infrastructure

UNESCO's initiative calls on the public sector to play a leading role in implementing open science (Das, 2021), and initiatives like the White House Office of Science and Technology Policy (OSTP) Memo on Advancing Open Science reinforce this mandate. International organizations play a crucial role in enabling open data sharing by fostering the necessary technical and social infrastructures; the Research Data Alliance (RDA) exemplifies this by building the vital bridges. Now is the time to develop AI for science cloud infrastructure to enhance equitable access to AI technologies, resources, and tools. Our recommendations are: (a) Establish an AI resource base aggregating computing power (e.g., GPU clusters), scientific datasets, pre-trained models, and software tools tailored to scientific research. This addresses common issues such as insufficient large-scale and high-performance computing capabilities and the scarcity of high-quality scientific datasets, and achieves the large-scale aggregation, high-efficiency allocation, and open sharing of AI public research resources. This infrastructure should be designed and operated in accordance with the FAIR Data Principles (Wilkinson et al., 2016) to maximize the discoverability, accessibility, interoperability, and reusability of its resources, particularly for both human researchers and automated systems. (b) Develop science foundation models and domain-specific models to enhance the underlying technology development and original innovation capabilities of AI for science and form modular and component-based applications that support secondary development (Moor et al., 2023). (c) Establish an open science cloud portal to provide scientific AI tools and intelligent agent services, research work space, and high-quality, integrated solutions and collaboration platforms for international big science research plans and projects as well as the global scientific community.

The development of an open ecosystem and comprehensive infrastructure for AI for science could impact scientific advancement as much as the late 20th-century Information Superhighway initiative impacted the development of the internet. Once necessary infrastructure is in place, an AI “moonshot” will become possible (PCAST, 2024). The U.S. NAIRR pilot exemplifies this direction, demonstrating potential through lowering access barriers to computing resources and datasets. However, its pilot scale limits reach, and integrating specialized tools and ensuring sustainable funding remain hurdles, partly due to cross-agency coordination complexity. Considering economic efficiency and sustainable development, it will be prudent to refurbish and upgrade existing open science cloud platforms as well as build new ones. For example, the AI4EOSC project enhances services with artificial intelligence for the European Open Science Cloud, facilitating its transition into the AI-driven era of open science (Vollmer, 2025).

### Bottom-up initiatives: popularizing open science across society

In 2023, the White House Office of Science and Technology Policy launched the “Year of Open Science” in collaboration with federal agencies, universities, and other organizations. This initiative is a high-profile and high-impact effort, but the “Year of Open Science” raised awareness at top levels; sustained cultural change across society and grassroots research communities remains limited, hindered by its short duration and insufficient mechanisms for long-term embedding. Initiatives should therefore also focus on the regularity and continuity of the push for universal awareness of open science.

Our recommendations are: (a) Advance popular science by encouraging scientists, AI experts, and science journalists to provide more and better popular science through websites, podcasts, and social media to effectively disseminate exemplary AI for science innovations and scientific achievements beyond academia and increase opportunities for public engagement (Fuentes, 2024). (b) Establish open courses covering spirits and practical knowledge of open science, fostering critical thinking and creativity to mitigate risks associated with overreliance on AI automation in innovation. (c) Promote the development of AI for science open-source communities like DeepModeling and the Generative Toolkit for Scientific Discovery, creating collaborative platforms for diverse disciplines and backgrounds to cultivate a collaborative, transparent, and inclusive open science culture. Ultimately, these bottom-up initiatives aim to make the transformative potential of scientific knowledge and AI tools accessible equitably across different races, ages, genders, and income levels and ensure that open science becomes a universal norm rather than an exception.

### Incentive policies

Our recommendations for incentive policies in industry are: (a) Conditionally open government-owned data repositories and international statistical databases (potentially involving enhanced privacy technologies and trusted research environments to improve access to sensitive data, or providing access through synthetic data methods). Similarly, high-quality scarce data from national laboratories, national large-scale scientific facilities, etc., could be opened for exchange, inviting reciprocal openness of private sector resources via central agreements to establish an open data network. (b) Provide incentives such as tax breaks, technology subsidies, and certification programs to guide industry participation in investment and development of AI for science infrastructure. (c) Promote collaboration between academia and industry by establishing national key R&D projects for AI for science, encouraging joint development of models and tools like AlphaFold (Jumper et al., 2021), GNoME (Merchant et al., 2023), GraphCast (Lam et al., 2023), with project proponents committing to open sharing of research outputs within AI for science infrastructure.

Our recommendations for incentive policies in academia are: (a) Encourage universities and research institutes to integrate activities such as managing public datasets, constructing large pre-trained science models, and related initiatives into performance assessments and promotion criteria for researchers to reward and recognize contributions to open science. (b) Encourage employers to establish roles such as AI infrastructure engineers, data pipeline specialists, and data openness managers, to contribute to the professionalization of open science management in the era of AI.

### Governance policies

Policy makers should systematically and comprehensively consider various dimensions of responsible artificial intelligence, such as reproducibility, interpretability, fairness, and transparency (Ahmed et al., 2023; Costes et al., 2024), in alignment with frameworks like the EU AI Act, when developing guidelines and regulatory principles for the AI era. Our recommendations are: (a) Promote open science practices in academic publishing by requiring open access to code, data, and computing environments (hardware, software, etc.) to address challenges of irreproducibility and ensure transparency in the research process or presentation of findings. (b) Prioritize methods to enhance model interpretability, such as integrating attention mechanisms, modular structures, and visualization tools, thereby improving reliability and transparency of models (Lam et al., 2023). (c) Emphasize the development of scientific benchmarks for evaluating large science models to provide a fair, open, and replicable standard for assessing model capabilities in scientific reasoning, knowledge extraction, and complex system simulations. Consider mandating industry to responsibly release model benchmarks, training data, validation procedures, etc., to prevent the development of closed ecosystems in the scientific domain. (d) Support the establishment of FAIR-aligned data pipeline standards to ensure data findability, accessibility, interoperability, and reusability throughout the research lifecycle, thereby mitigate issues like data bias, privacy breaches, and model hallucinations. This should be reinforced by national legislation mandating open access for publicly funded research data, exemplified by France's Digital Republic Law (Loi République numérique). (e) Strengthen collaboration between AI experts, scientists, and social experts from legal, psychological, and public administration fields to study the interaction between open science and AI for science, explicitly addressing ethical risks such as algorithmic bias exacerbating scientific inequality and proprietary barriers impeding knowledge-sharing. This collaboration should minimize potential security and ethical risks while ensuring clear channels for public oversight.

The novelty of our proposal lies in a pioneering “vertical-horizontal” dual-track framework for AI-driven open science, integrating: Vertical coherence bridging top-down AI infrastructure (e.g., NAIRR) with bottom-up cultural cultivation (e.g., open science initiatives); Horizontal synergy coupling incentive accelerators (tax benefits, data reciprocity) with governance stabilizers (refer to EU AI Act); Cross-layer permeation embedding FAIR principles throughout infrastructure/governance to resolve implementation gaps in cross-agency coordination, sustained adoption, and tool integration—strategically advancing EU Open Science Policy objectives.
